# Non-Protein Thiol Compounds and Antioxidant Responses Involved in Bryophyte Heavy-Metal Tolerance

**DOI:** 10.3390/ijms24065302

**Published:** 2023-03-10

**Authors:** Giovanna Salbitani, Viviana Maresca, Piergiorgio Cianciullo, Rosanna Bossa, Simona Carfagna, Adriana Basile

**Affiliations:** Department of Biology, University of Naples Federico II, 80138 Naples, Italy

**Keywords:** antioxidant enzymes, bryophyte, heavy metals, glutathione, phytochelatins, stress responses, metal tolerance

## Abstract

Heavy-metal pollution represents a problem which has been widely discussed in recent years. The biological effects of heavy metals have been studied in both animals and plants, ranging from oxidative stress to genotoxicity. Plants, above all metal-tolerant species, have evolved a wide spectrum of strategies to counteract exposure to toxic metal concentrations. Among these strategies, the chelation and vacuolar sequestration of heavy metals are, after cell-wall immobilization, the first line of defence that prevent heavy metals from interacting with cell components. Furthermore, bryophytes activate a series of antioxidant non-enzymatic and enzymatic responses to counteract the effects of heavy metal in the cellular compartments. In this review, the role of non-protein thiol compounds and antioxidant molecules in bryophytes will be discussed.

## 1. Introduction

Heavy metals are a group of elements with metallic properties (e.g., transition metals, metalloids, lanthanides, actinides) having an atomic mass greater than 20 and a gravity greater than 5 g·cm^−3^ [1,2]. Fifty-three elements fall into this category, and some examples are copper (Cu), tin (Sn), iron (Fe), cobalt (Co), zinc (Zn), cadmium (Cd), mercury (Hg), and lead (Pb). Among heavy metals, some are essential for living organisms (e.g., Zn, Cu, Fe, Co, etc.) whilst others are essentially toxic and cause harmful effects on the organisms (e.g., Hg, Cd, Pb, As, etc.) [3] Naturally, heavy metals are present in the environment and are vital for the survival of all organisms, but they may become hazardous when they accumulate inside them [4]. Over the last decades, heavy metal pollution has become a threat to the environment and human health. The contamination has been observed in soil, water and air. The cause is mainly attributed to anthropogenic activities such as mining, industrial production, and the use of metal-containing compounds in domestic and agricultural settings [3]. Conversely, lithogenesis, weathering, erosion, and other geological processes are the natural sources [5]. Unfortunately, heavy metal contamination is widely distributed, and persists long-term [6]. Data from several studies report that the annual worldwide release of heavy metals is about 22,000 ton for Cd, 939,000 ton for Cu, 783,000 ton for Pb and 1,350,000 ton for Zn [7,8]. According to the Environmental Protection Agency (EPA), arsenic, cadmium, lead, and mercury, are among the most hazardous metals in the environment [9]. In plant and animal cells, heavy metals have been reported to damage cellular organelles and components such as cell membrane, mitochondrial, endoplasmic reticulum, and nuclei [10]. Metal ions have been found to interact with DNA and nuclear proteins, causing genotoxicity and negative conformational changes [3]. Hyperaccumulator plants are able to survive and grow at contaminated sites having high metal concentrations, and accumulate metals beyond the maximum threshold levels [8]. Among bryophytes, there are species that are known as hyperaccumulator plants [11,12]. The bryophytes represent the most conservative group of land plants [13]. They were the first plants colonizing the land, and as such had to develop mechanisms to cope with the much greater amounts of metals present in the environment [14]. Tracheophytes have developed a series of histological-anatomical adaptations to limit the entry of heavy metals (i.e., a strong cutinization of the leaves, the limitation of exchanges at the stomatal level, protection of the stems with suberin, and endodermis compartmentalization at the root level, etc.), while bryophytes, not having these anatomic adaptations, have developed cellular responses that have allowed them to survive in polluted environments. These characteristics resulted in the ability of bryophytes to be consistent colonizers of metal-contaminated places or to accumulate large amounts of metals without any evident negative effect [15,16]. In general, plant cells employ various strategies to survive and cope with the toxic effects of heavy metals [17]. Chelation is one of the strategies of plants for this purpose [16,18]. This mechanism also operates in bryophytes, as has been demonstrated by numerous studies [16,19,20,21,22]. Furthermore, heavy metals can generate oxidative pressure in the plant cell, with the consequent toxicity. In this context, several antioxidant mechanisms have been observed in bryophytes, such as changes in antioxidant activities for scavenging the Reactive Oxygen Species (ROS) in different compartments inside plant cells.

The focus of this review is to summarize the toxic effects of heavy metals and recent developments on the role of non-protein thiols in metal tolerance in bryophytes.

## 2. Bryophytes and Heavy Metals

### 2.1. Metal Tolerance and Accumulation of Heavy Metals in Bryophytes

Bryophytes lack an absorptive root system, have a cuticle endowed with high permeability and exhibit a pronounced cell-wall ion-exchange capacity, all of which enables them to efficiently absorb minerals across their entire body surface [23]. This property is a fundamental adaptive factor for most, if not all, bryophytes, but, at the same time, it results in high sensitivity to toxic chemical species present as contaminants in the environment. In the presence of the same elements and environmental conditions, bryophytes can exhibit different types of behaviour, with different biochemical mechanisms allowing them to tolerate high concentrations of heavy metal. Metal tolerance is the ability of a species to survive in environments where metal(loid)s contents are toxic for most other species [24]. It can be a constitutive property (genetically determined), or induced as a result of exposure to heavy metals, or mixed in nature [25]. For example, Basile et al. [11] found only the moss *Funaria hygrometrica* growing on the soil of lead and zinc mine dumps, which probably represented an ecotype with a high tolerance to such prohibitive environmental conditions [11]. In a subsequent study, Basile et al. [26] showed that spores of *F. hygrometrica* collected from a metal-polluted site developed in a normal protonemata, whilst those from an unpolluted site grew in an altered protonemata. Comparably, Jules and Shaw [27], observed that protonemata developed in vitro from samples of *Ceratodon purpureus* growing near a smelter were more tolerant to Zn, Cd and Pb than those from unpolluted sites. Constitutive metal tolerance is stable and unaffected by environmental conditions; induced metal tolerance, being a form of physiological acclimatization to the environment, persists as long as the specific stressors that led to its onset exist [28]. In most cases, tolerance appears to be genetically determined rather than induced, and selection for tolerance can be severe in highly contaminated habitats [28,29].

The metal tolerance in bryophytes has been investigated in several studies. Populations of the same species that colonize urban environments might have been selected for tolerance to Pb, as observed by Briggs et al. [30], comparing *Marchantia polymorpha* from unpolluted and polluted urban environments. Similarly, Wells and Brown, 1995 [31] compared two populations of *Rhytidiadelphus squarrosu*, one collected from an unpolluted site and the other from a zinc mine. Samples from a contaminated site showed a lower loss rate in photosynthetic activity than those from an unpolluted one. The existence of metal-tolerant ecotypes in bryophytes was observed in several other studies [32,33,34]. Patterns of heavy metal uptake and accumulations in bryophytes have been investigated both in mosses and liverworts. Basile et al. [35] characterized uptake and localization of Pb in *Funaria hygrometrica*. The results showed that Pb was accumulated preferentially in some parts of the gametophyte and in the lower parts of the sporophyte. On the other hand, no Pb was detected in the spores and capsule (i.e., upper part of the sporophyte). Basile et al. [11] confirmed the previous results analysing Pb and Zn content in *F. hygrometrica* collected from a mine-tailing site. The authors observed that Pb and Zn were mostly accumulated into the gametophytes (1000- to 2000-fold more) than in the sporophyte. A few years later, Carginale et al. [36] investigated the accumulation and localization of Cd in the liverwort *Lunularia cruciata*. The results showed that Pb accumulated mainly in the hyaline parenchima (i.e., non-photosynthetic tissue with large vacuoles) and was sequestered into vacuoles. These data suggest that bryophytes tend to avoid the accumulation of heavy metals in the reproductive structures (i.e., sporophyte, spores, and gemmae), and sequester them into the vacuole of the gametophyte cells.

### 2.2. Bryophytes’ Defences against Heavy Metals

Heavy metals, especially those which do not have a role in bryophytes’ physiology (e.g., Pb, Cd, Hg), cause harmful effects, starting from the cellular level, which may cause physiological impairments in the whole organism. This happens when the molecular machinery cannot manage the excess of heavy metal in the cytoplasm. Several studies have investigated the harmful effects of heavy metals in bryophytes. Some researchers have characterized the damage at the ultrastructural level. Basile et al. [12] and Esposito et al. [37] reported that metals such as Cd and Pb (Cd > Pb) cause severe alterations in the cell ultrastructure. The authors observed dose-dependent alterations: swollen chloroplasts, irregular thylakoids organization, increased plastoglobules, swollen mitochondria cristae; and cellular signs of senescence (i.e., multivesicular bodies). Similar alterations were observed by Choudhury and Panda [38,39], in the moss *Taxithelium nepalense* after Pb and As exposure. Other studies pointed out the fact that heavy metal uptake causes a decrease in chlorophyll content [40,41,42,43,44], and a decrease in photosynthetic activity [45,46,47,48]. These changes at the cellular level cause toxicity at the organism level due to alteration of the normal metabolism. In fact, some other investigations reported growth inhibition of bryophytes exposed to toxic metals such as Pb and Cd [43,49,50,51].

The metal tolerance in bryophytes could have an explanation in precise cellular responses such as the activation of certain enzymes and the synthesis of defence proteins.

As in other plant organisms, in bryophytes, the first barrier against heavy metal stress is mediated by the cell wall through chelation and immobilization via pectic compounds. Several studies have investigated the immobilization of heavy metals in the cell walls of bryophytes [11,35,36,52,53]. This passive mechanism reduces the amounts reaching young or reproductively affected parts and, at the cellular level, the amounts able to penetrate the cytoplasm and exert toxic effects. Heavy metals bind to the negative charges of cell-wall polysaccharides rich in carboxyl groups (homogalacturonans) and other functional groups (–OH and –SH), as well as proteins, phenolics, and amino acids [54,55]. This process mainly affects tissues that exhibit cell-wall modifications, such as hydroids, placental “transfer cells”, or hyaline parenchyma cells. This process seems to be increased by stress conditions. In fact, the break-up of cell membrane in dead or damaged cells may cause the freeing of more cation-binding sites, thus allowing a higher accumulation of metals in the cell wall of dead or damaged cells [56]; there is proof that bryophytes under heavy metal stress can rearrange the cell wall by thickening it and increasing the amount of low-esterified and unesterified homogalacturonan [57,58]. In general, these mechanisms aim to provide more binding sites for the immobilization of heavy metals in the cell wall.

**Figure 1:**
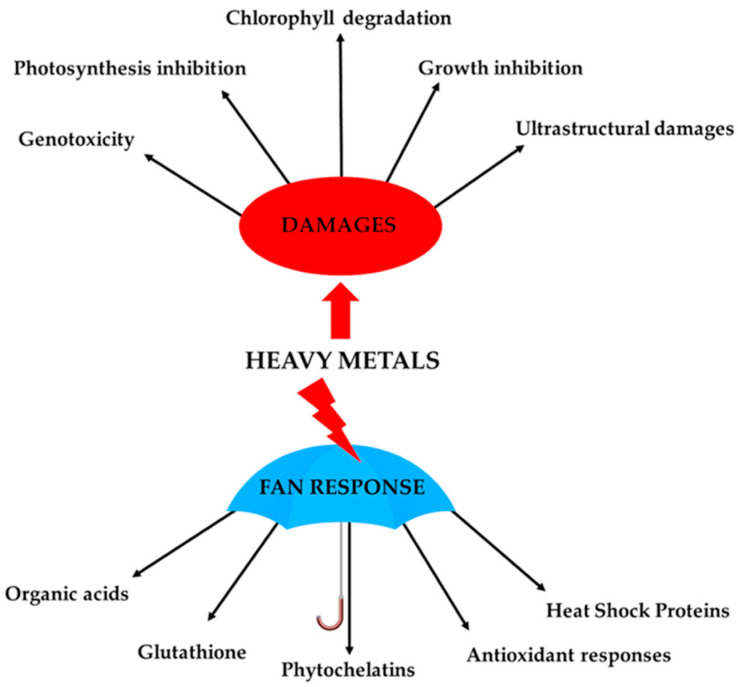
Schematic figure of cellular responses and alterations caused by heavy metals in bryophytes.

The cell wall thus represents a passive barrier to prevent heavy metals from entering into and interacting with the cytoplasmic environment. However, heavy metals that enter the cytosol require a wide range of molecular responses to avoid harmful effects to cellular structures. Bryophytes have developed, like higher plants, a series of cellular responses to counteract heavy metal stresses that collectively take the name “fan response” (Figure 1). These cellular mechanisms include the chelation and compartmentalization of heavy metals, as well as the activation of non-enzymatic and enzymatic antioxidant defences to counteract the induced reactive-oxygen-species (ROS) production. Several studies have indicated that these mechanisms involve the synthesis of molecules capable of binding such ions (e.g., amino acids, citric acid, malic acid) [59,60], the modulation of the enzymatic antioxidant system (i.e., SOD, CAT, GPX, POX, etc.) [38,45,61,62,63,64], increase in the phenolic content [65], increase in lunularic acid synthesis [65], and increased synthesis of phytochelatins, glutathione and “heat shock protein” (HPS) [37,66,67,68,69,70], phenomena largely mediated by gene activation/repression (Figure 1).

### 2.3. Non-Enzymatic and Enzymatic Antioxidant Systems in Bryophytes

**Figure 2:**
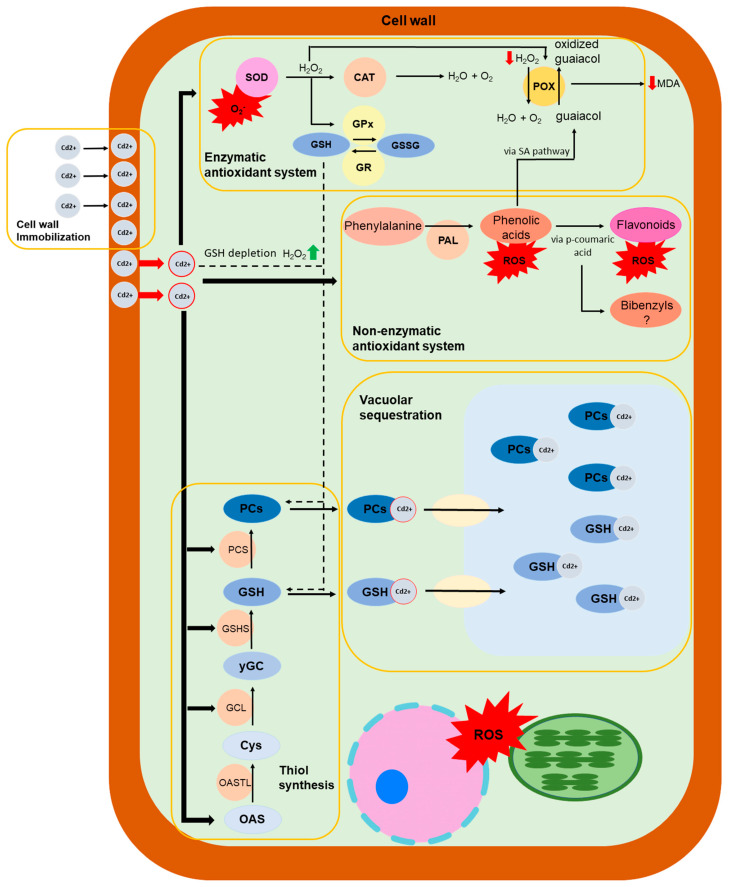
Schematic diagram of the mechanisms involved in metal tolerance in bryophytes. Cadmium is selected as an example specimen. List of abbreviations: SOD (superoxide dismutase), CAT (catalase), GPx (glutathione peroxidase), GR (glutathione reductase), POX (guaiacol peroxidase), PAL (phenylalanine ammonia lyase), OASTL (O-acetylserine (thiol) lyase), GCL (glutamate-cysteine ligase), GSHS (glutathione synthetase), PCS (phytochelatin synthetase), Cys (cysteine), OAS (O-acetylserine), yGC (y-glutamylcysteine), GSH, (reduced glutathione), GSSG (oxidized glutathione), PCs (phytochelatins), MDA (malondialdehyde), ROS (Reactive Oxygen Species).

Since glutathione is also involved in the antioxidant response (Figure 2), it is worth mentioning studies that have investigated the non-enzymatic and enzymatic antioxidant systems in bryophytes. Heavy metals can cause an overproduction of ROS, and for this reason bryophytes must possess an efficient antioxidant system to cope with ROS-induced oxidative stress (Figure 2). This antioxidant system is essential to maintain cellular redox homeostasis in plants. This system includes numerous enzymes and low-molecular-weight compounds [71].

The protection of cells against heavy-metal-induced oxidative stress can occur via non-enzymatic antioxidant systems (Figure 2). Non-enzymatic antioxidants include hydrophilic (ascorbate, glutathione), lipophilic (α-tocopherol and carotenoids), phenolic, and flavonoid small molecules.

Glutathione and ascorbate are compounds implicated in redox signal transduction, acting as second messengers in hormone-mediated responses [72]. α-tocopherol is a fat-soluble antioxidant belonging to the vitamin E class, and is involved in the protection of cell membranes from the effects of ROS. Flavonoids, an important class of polyphenols that perform multiple functions in plants, appear to be cofactors of enzymatic action and antioxidant activity; their biosynthesis is stimulated in the presence of stress. Carotenoids are also important molecules in plant defence, as they act as negative regulators of oxidative stress; they are also able to interact synergistically with the other antioxidants, thus enhancing the protection of the plant.

In Maresca et al. [65] non-enzymatic antioxidant-defence enhancement was observed as an increase in the total phenolic content and lunularic acid synthesis in the liverwort *C. conicum* exposed (7 days) to a mix of heavy metals such as Cu, Zn, Pb and Cd. Lunularic acid is a bibenzyl, an abundant group of molecules in liverworts [73,74], which serve as the precursor molecule for the synthesis of other bibenzyls and bis-bibenzyls. The synthesis of lunularic acid starts from phenylalanine [75]. Thus, this compound might be a by-product of the activation of the flavonoid biosynthesis via the phenylpropanoid pathway [76] or be actively involved in the antioxidant response. To date, the role of bibenzyls in stress response to heavy metals has not yet been ascertained. Other studies have demonstrated that, as in other plants [71], ascorbic acid may play a role in the non-enzymatic antioxidant response against heavy metal stress in bryophytes, participating in both short- and long-term response. Accumulation of ascorbate was observed in *H. plumaeforme* with different concentrations of Pb and Ni (48 h) [77,78], in *Taxiphyllum nepalense* exposed to Pb and Cr [38], and in *Taxiphyllum barbieri* after a short exposure (24 h) to Cd [59]. Similar results were obtained with the protonemata of the model moss *Physcomitrium patens* after a long-term exposure (40 days) to Cd [60]. Furthermore, the authors observed an accumulation of citrate and malate, which could have acted as chelating agents against Cd.

The enzymatic components of the antioxidant defence system include the superoxide dismutase (SOD) [79,80], catalase (CAT) [79,81], glutathione peroxidase (GPx), glutathione reductase (GR) [82], guaiacol peroxidase (POX) [83], and peroxiredoxins (Prxs) [84,85]. Several studies have demonstrated that enzymes involved in the antioxidant defence vary among the species and depend on the specific metal element. For example, Sun et al., 2009 and 2010 [77,78] treated the moss *H. plumaeforme* with different concentrations of Pb and Ni, singly or combined. They found that peroxidase was the main enzyme active in counteracting the resulting oxidative stress with a dose-dependent response. SOD enzyme activity increased only slightly, and CAT activity actually decreased. Aydoğan et al. [86] evaluated the impact of Pb, Ni, Cu and Cr oxidative stress in two bryophyte species, *Pleurochaete squarrosa* and *Timmiella barbuloides*. Cu treatment induced SOD activity in *P. squarrosa*, but not in *T. barbuloides*. Similarly, increased SOD activity was observed in *Fontinalis antipyretica* under Cu exposure [61] and in *T. nepalense* under Cr exposure [38]. The opposing results of SOD activity in response to Cu and Cr between the two species could be due to possible differences in the redox state of the cells. The study by Aydoğan et al. [86] showed that exposure to Ni, Pb and Cr did not affect CAT activity in *P. squarrosa* and *T. barbuloides*. Conversely, Choudhury and Panda [38] demonstrated that treatment with Pb and Cr of *T. nepalense* caused a decrease in CAT in short-term exposure. Sun et al. [77] also evaluated how Pb and Ni treatment of *Hypnum plumaeforme* caused a decrease in CAT activity. Therefore, this could suggest that CAT activity in response to Ni, Pb and Cr is strongly based on the specie’s detoxification mechanisms and the retention capacity of these metals in the cell walls. However, Cu is a redox-active metal and a known catalase inhibitor, and thus is able to suppress CAT activity and cause oxidative stress in cells [87]. POX activity induced by toxic concentrations of Pb and Ni has been reported as a common response in various bryophyte species, such as *Hypnum plumaeforme, Thuidium cymbifolium* and *Brachhythecium piligerum* [88]. However, the Cu induced POX activity in *P. squarrosa* but suppressed it in *T. barbuloides*. Cr-treated *T. barbuloides* samples accumulated excess H_2_O_2_ in correlation with suppressed POX activity [86]. The data agree with the results of decreased POX activity in the bryophyte *T. nepalense* [38] and *Taxiphyllum taxirameum* [89] exposed to Cr. The discrepancy in POX activity in moss species indicates that different enzymatic (CAT, APX) and non-enzymatic scavengers operate in the elimination of H_2_O_2_ from cells.

The activity of antioxidant enzymes (CAT, SOD, GST and POX) was found to be increased in the aquatic moss *Leptodictyum riparium* by Maresca et al. [62] after the exposure to a mixture of metals (Cd, Cu, Pb, Zn). This study showed that the enzymatic activities followed a metal-concentration-dependent increase. Similar results were observed in *Conocephalum conicum* under cadmium stress [64]. These results agree with those obtained by Bellini et al. [22], who studied the effects of Cd on the enzymatic activity of *L. riparium*. Dazy et al., 2009 [61] studied the activity of GR in *F. antipyretica* exposed to Cd, Cu, Pb, and Zn. In particular, after Cu exposure, a bell-shaped concentration-response trend in GR was observed, but only if the exposure was at 0.1 M. In contrast, regardless of whether the moss was exposed to Cd, or Zn, or Pb, no bell-shaped concentration-response trends were observed following exposure; indeed, GR activity remained unchanged.

The same authors [90] found that chromium induced GR activity in the moss *F. antipyretica;* as for as the effect of chromium, the increase in the activity of GR in the moss *F. antipyretica* subjected to stress from Chromium III (tested in the form of both chloride and nitrate) and Chromium VI, was studied. In particular, GR was induced by a low level of Cr(NO_3_)_3_, (i.e., 6.25 × 105 mM), while exposures to CrCl_3_ and K_2_Cr_2_O_7_ induced significant responses only after the addition of 25 and 50 mM, respectively. Moreover, GR was highly correlated with APX in the chromium VI experiment (R = 0.81; *p* = 0.004). The increase in GR activity upon Cr exposure was explained by a higher cellular consumption of reduced GSH resulting from at least two putative mechanisms: (i) an increase in the glutathione-ascorbate cycle rate in order to detoxify ROS; (ii) an incorporation of GSH into the unidentified thiol compounds.

Furthermore, in the same work, the observed relationships between cell damage (malondialdehyde (MDA) level) and antioxidant enzymes suggested that the tolerance of *F. antipyretica* for heavy metals it depended at least in part on its ability to prevent oxidative action. In addition, comparing the toxicity of each heavy metal, significant increases in the at MDA level was determined from lower concentrations (since 10 µM was observed) with non-essential metals.

However, a significant role in heavy metal tolerance and detoxification is played by vacuolar compartmentalization, which prevents the circulation of free metal ions into the cytoplasm, where they might interfere with key metabolic processes. The process of intracellular heavy-metal chelation and sequestration within the vacuole is accomplished by thiol-compounds such as glutathione and phytochelatins.

### 2.4. Non-Protein Thiol Compounds in Heavy Metal Stress

Non-protein thiols is a term that includes all low-molecular-weight thiol compounds containing a sulfhydryl group (-SH) in their structure [91]. These compounds can be found in most plants, microorganisms and all mammalian tissues [92]. They are considered as antioxidants that work through a variety of mechanisms: (i) as the components of the general thiol/disulfide redox buffer, (ii) as metal chelators, (iii) as radical quenchers, (iv) as substrates for specific redox reactions, and (v) as specific reductants of individual protein disulfate bonds [93]. In several plant species, they exist under various forms: (i) the tripeptide: glutathione (γ-glutamylcysteinyl glycine), (ii) homoglutathione (γ-l-glutamyl-cysteinyl-β-alanine) which can replace entirely or partially glutathione and (iii) polymerized peptides, phytochelatins or homophytochelatins. These molecules are synthesized by enzymatic polymerization (by γ-glutamyl-cysteine synthase) of glutathione or homoglutathione [92,94]. They play a key role in the regulation of redox balance and can be used as indicators of oxidative stress in the detoxification process against xenobiotics and heavy metals [92,95]. The influence of non-protein thiol upon heavy metals is due to their extremely high affinity for SH residues [92,96,97]. The most abundant low-molecular-weight thiol is the glutathione (GSH), which is synthesized from Cys.

### 2.5. Cysteine Biosynthesis

Cysteine (Cys) is a polar amino acid and a chiral molecule; its side group has a thiol functional group (-SH). Cys synthesis represents the final step in assimilatory sulfate reduction and the process by which reduced sulphur is taken up for metabolism not only of plants, but also for the human food chain in general [98,99]. Therefore, Cys is an important metabolite, as it serves as a sulphur donor for the synthesis of Methionine, iron-sulphur clusters, some vitamins, such as thiamine and biotin, of lipoic acid and coenzyme A, GSH and thiol-containing proteins [100].

The Cys synthesis pathway in plants occurs almost constitutively and ubiquitously in the plastids, the cytosol and the mitochondria [100]. It starts with serine acetyltransferase (SAT; EC 2.3.1.30) and the activation of serine by acetyl transfer from acetyl coenzyme A to form O-acetylserineo-acetylserine (Figure 2) [100]. Then, the o-acetylserine (thiol) lyase (OASTL, EC 4.2.99.8) substitutes the acetate residue with reduced sulphur (H_2_S), to form Cys [99].

The mean free-Cys concentration in unstressed plant cells is low (10–30 μM) [98]; however, studies reported an increase in free-Cys levels in response to different abiotic stresses [99]. Numerous studies have reported the increase in free Cys together with the GSH content, which has led to the conclusion that Cys is mainly needed for the biosynthesis of sulphur-rich compounds such as GSH and stress-related proteins [101,102].

### 2.6. Glutathione

GSH was first identified in yeasts in 1888, but its structure was not described until 1935. Intensive study began in the 1960s, due to the discovery of its functions in human body fluids. The clarification of GSH metabolism was credited to Dr. Alton Meister, due to his indisputable contribution [103,104].

GSH is a low-molecular-weight thiol tripeptide constituted of glutamate (Glu), cysteine (Cys), and glycine (Gly) [8,9,10]. In plant cells, it was found in the different organelles, and it has been determined to have millimolar concentrations (0.08–15 mM) which differ markedly among plant species [105,106,107,108]. In *Arabidopsis* leaf tissue, glutathione was measured in mitochondria (15 mM), nuclei (6.4 mM), cytosol (4.5 mM), peroxisomes (4.4 mM), chloroplasts (1.2 mM) and vacuole (0.08 mM) [107]. Glutathione exists either in a reduced form (i.e., GSH) with a free thiol group or in an oxidized form (i.e., GSSG) with a disulphide bond linking two glutathione molecules [105,109]. GSH plays an important role in the regulation of developmental processes such as cell division [110,111], flowering [110,112], and in protecting plants against abiotic stresses such as nutritional starvation, heavy metal exposure, drought, salinity, heat, cold, and certain exogenous and endogenous organic-chemical agents [16,110,113,114]. GSH is also a major transport and storage form of reduced sulphur in plants [16].

The pathway of GSH synthesis from its constituent amino acids involves two ATP-dependent enzymes, as in animals (Figure 2) [105,106,110]. The first enzyme, γ-glutamylcysteine synthetase (GSH1, E.C. 6.3.2.2) catalyzes the formation of a peptide bond between the carboxyl group of glutamate and the amino group of Cys, to form γ-glutamylcysteine (γ-EC) in chloroplasts and in plastids [105,106,110,115]. The second enzyme, glutathione synthetase (GSH2, E.C. 6.3.2.3), in contrast to γ-ECS, which is localized only in plastids, is found in both the plastids and cytosol [106,116] and it ligates a Gly residue with γ-EC to form GSH [105,106,110].

Gamma-glutamylcysteine and GSH are transported across the bounding envelope membranes of the chloroplasts by a small family of transporters called the chloroquine-resistance-transporter-like transporter (CLT:37). Thereafter, GSH is transported to all intracellular compartments including the nucleus [106]. GSH levels can be regulated in two different ways: (i) at the level of gene transcription [93,106], and (ii) under stress conditions, at the level of γ-ECS activity, through thiol-based oxidative activation or feedback inhibition by GSH [93,106].

Since sulfur levels modulate Cys content in plants, a clear consequence is that GSH levels can be affected by both the availability of sulphur and Cys, as well as Gly in some circumstances [105,117].

During metal stress, GSH plays a crucial role in chelation. In fact, GSH (i) may be directly involved, because its thiol group is susceptible to different metals, such as Hg and Cd; (ii) can mitigate the redox imbalance caused by toxic-metal accumulation through its antioxidant power; and (iii) may be indirectly involved as a precursor of phytochelatins (PCs), ligand peptides having a particularly high affinity for some heavy metals [16,19,113]. In particular, the nucleophilic nature of the thiol group is involved in the formation of mercaptide bonds with metals and in reacting with selected electrophiles [110]. This reactivity makes GSH a suitable biochemical to protect plants against heavy metal exposure.

Several studies show that exposure of plants to high levels of heavy metals induces ROS formation, either directly or indirectly, by influencing metabolic processes [93,110,118,119]. ROS are reactive oxygen-containing molecules possessing an unpaired electron. Under standard growth conditions, ROS levels in a plant cell are tightly controlled by the ROS-scavenging systems, which include GSH (Table 1). In particular, GSH participates in the control of the H_2_O_2_ level of plant cells through glutathione peroxidase (GPx) [110,118,119]. However, when ROS levels overcome the capability of the antioxidant system, then oxidative stress occurs [110]. The changes in the ratio between reduced GSH and oxidized GSSG is important in the redox-signalling pathways during the detoxification of H_2_O_2_ [120]. Indeed, the GSH/GSSG ratio serves as monitor of the redox state of the cells, and is involved in ROS perception [120,121].

Moreover, GSH has a direct chelating action against heavy metals. The conjugation between GSH and heavy metals is operated by the glutathione S-transferase [106,110,115]. After the conjugation, the GSH-metal complexes are transferred to the vacuole, avoiding the interaction with other intracellular compartments [110,122]. However, the massive use of reduced GSH results, at least temporarily, in a decrease in its cytosolic level [110,123]. This effect could directly influence the GSH/GSSG redox potential, generating a redox signal in stress-exposed cells [124]. Consequently, any massive involvement of GSH in the detoxification processes will impact cellular redox poise. Therefore, under such circumstances, the maintenance of the GSH/GSSG ratio becomes crucial for the survival of plants [110].

**Table 1:** Significant studies on the role of glutathione and phytochelatins in heavy-metal-stress tolerance in bryophytes.

Organism	Heavy Metal	[mM]	Vivo	Vitro	Glutathione	Phytochelatins	Ref.
*Lunularia cruciata*	Cd	0.0008 mM		X	-	-	[36]
		0.004 mM				-	
		0.002 mM				-	
*Lunularia cruciata*	Cd	0.036 mM	X	X	14.4 ± 3.2 nmol g^−1^ fw	11.2 ± 0.7 nmol g^−1^ fw	[14]
	Fe	0.1 mM			-	-	
	Zn	0.1 mM		X	-	-	
*Lunularia cruciata*	Al, As, Cd, Cu, Cr, Fe, Pb, Mn, V, Hg, Ni	different concentrations in the three sites studied	X		0.2 ± 0.02 nmol g^−1^ fw	3.7 ± 0.1 nmol g^−1^ fw	[63]
*Sphagnum palustre* L.	Cd	0.036 mM		X	34.2 ± 2.1 nmol g^−1^ fw	13.3 ± 0.8 nmol g^−1^ fw	[125]
*Polytrichastrum formosum*					367.7 ± 25.6 nmol g^−1^ fw	N.D	
*Hypnum cupressiforme*					123.6 ± 28.1 nmol g^−1^ fw	N.D	
*Fontinalis antipyretica*					148.0 ± 19.1 nmol g^−1^ fw	N.D	
*Leptodictyum riparium*	Cd	0.036 mM		X	-	50.89 ± 3.88 pmol PCn g^−1^ fw min^−1^ (PCs activity)	[22]
		0.36 mM		X	-	96.83 ± 5.77 pmol PCn g^−1^ fw min^−1^ (PCs activity)	
		0.1 mM		X	-	90.77 ± 6.57 pmol PCn g^−1^ fw min^−1^ (PCs activity)	
*Marchantia polymorpha* L.	Cd	50 mM		X	-	-	[126]
	Zn	0.2 mM		X	-	-	
*Nitella mucronate*	Cd	0.018 mM		X	-	22.6 ± 4.5 nmol g^−1^ fw	[127]
	Cd	0.036 mM		X	-	33.8 ± 9.0 nmol g^−1^ fw	
	Fe(II)	0.0075 mM		X	-	18.5 ± 1.3 nmol g^−1^ fw	
	Fe(II)	0.03 mM		X	-	12.7 ± 1.6 nmol g^−1^ fw	
	Fe(III)	0.0075 mM		X	-	19.6 ± 1.0 nmol g^−1^ fw	
	Fe(III)	0.03 mM		X	-	40.1 ± 6.2 nmol g^−1^ fw	
	Zn	0.030 mM		X	-	7.1 ± 3.7 nmol g^−1^ fw	
*Fontinalis antipyretica*	Cr	From 6.25 × 10^−5^ to 50 mM		X	6.25 × 10^−2^ mM	-	[90]
*Fontinalis antipyretica*	Cd	0.1 mM		X	1.95 mM	-	[128]
*Fontinalis dalecarlica*	Cd	0.1 mM		X	0.42 mM	-	
*Taxithelium nepalense*	Cr	0.1 mM		X	-	-	[38]
	Cr	1 mM		X	-	-	
	Pb	0.1 mM		X	-	-	
	Pb	1 mM		X	-	-	
*Polytrichum comune*	Cr	0, 0.01 mM		X	-	-	[129]
	Cr	0.1 mM		X	-	-	
	Cr	1 mM		X	-	-	
	Cu	0, 0.01 mM		X	-	-	
	Cu	0.1 mM		X	-	-	
	Cu	1 mM		X	-	-	
	Zn	0, 0.01 mM		X	-	-	
	Zn	0.1 mM		X	-	-	
	Zn	1 mM		X	-	-	
*Physcomitrella patens*	Cd	0.005 mM		X	226.2 ± 42.9 nmol g^−1^ fw (24 h after the treatment)	-	[130]
					286.9 ± 51.7 nmol g^−1^ fw (72 h after the treatment)	-	
					369.3 ± 44.1 nmol g^−1^ fw (120 h after the treatment)	-	
	Cd	0.01 mM		X	240.6 ± 10.6 nmol g^−1^ fw (24 h after the treatment)	-	
					354.5 ± 27.0 nmol g^−1^ fw (72 h after the treatment)	-	
					425.5 ± 1.3 nmol g^−1^ fw (120 h after the treatment)	-	

X = select study in vivo or in vitro; N.D = not detected.

### 2.7. Phytochelatins

Phytochelatins (PCs) are a set of heavy-metal-binding peptides which consists mainly of the amino acids Glu, Cys, and Gly, where the latter two are linked and result in the formation of g-Glu-Cys dipeptides [110,131]. PCs were discovered first by Hayashi and his group (1981) [132] as Cd-binding complexes in fission yeast, *Schizosaccharomyces pombe*, exposed to Cd_2_^+^, and were named as “cadystins” [132,133,134], and then in cell suspension cultures of the higher plant *Rauvolfia serpentina* after exposure to Cd [126,131,135]. Afterwards, PCs were identified in all plant species, and investigated as well as in algae, fungi, and diatoms [126,136]. PCs are synthesized inductively by exposure not only to Cd, but also to other heavy metals such as Hg, Cu, Zn, Pb and Ni [110,131]. Glutathione, hydroxymethyl-glutathione, and G-glutamylcysteine were reported as the major precursors for PC synthesis [131,137,138] through the enzyme PC synthase (PCS) (Figure 2); the activation of this enzyme requires a post-translational modification which can be induced by the toxic metals [131,138]. PC synthase catalyses the transpeptidation of the γ-Glu-Cys moiety of GSH either onto a second GSH molecule to form PC_(*n*=2)_ or onto a PC molecule to produce a PC_(*n*+1)_ oligomer [139].

The function of PCs is mainly reflected in two aspects: improving plant resistance to heavy metals and heavy-metal chelation, to detoxify the plant cells [134]. In particular, the main function of PCs is the ability to carry out metal complexation. PC-metal complexes are one of the ways to improve the resistance of plants to heavy metals, which proves that the roles of PCs are to protect plants against toxic metals [140]. The formed complexes between the heavy metal ions and PCs can then be transported into the vacuole, decreasing the concentration of metals in the cytoplasm and protecting the plants from their deleterious effects [16,110].

The functional relevance of PCs in bryophytes was addressed only recently (Table 1). While clear biochemical evidence of PC activity in liverworts has been clarified [141,142], only one species of moss investigated so far (*Sphagnum palustre* L.) showed sustained PC production [125,142]. However, no PC gene is present in the genome of the model moss *Physcomitrium patens* Mitten, indicating that other metabolic pathways may play major roles in metal detoxification in mosses [141]. This is supported by the fact that the highly-cadmium-tolerant moss *L. riparium* produces only trace amounts of PCs [22], and the single-copy PC gene present in the genome of *M. polymorpha* has been isolated and functionally characterized by overexpression in heterologous systems, demonstrating that it is enzymatically active and able to complement the *Arabidopsis* knockout cad1–3 mutant [126]. However, in the lack of stable *M. polymorpha* PCS knockouts, the outstanding questions of whether PC-mediated detoxification plays a role as pivotal in liverworts as that in angiosperms, and its relative relevance with respect to other detoxification pathways, have remained unanswered. This is particularly important, considering the relevance of bryophytes as bioindicators of metal [20].

### 2.8. Glutathione and Phytochelatins in Bryophytes

Despite their apparent structural simplicity, bryophytes have developed a set of different strategies to tolerate pollution and in particular heavy metal stress [20]. In bryophytes, the cuticle, roots, and transport system are not fully developed, so nutrients and pollutants such as heavy metals pass directly through the leaf surface into the cytoplasm [19].

Glutathione and/or phytochelatins are directly involved in the fight against heavy metal stress in bryophytes. Under exposure to heavy metals, they can be chelated by GSH and PCs and then transported into vacuoles via ABC-like transporters [39], decreasing, in this way, the concentration of metals in the cytoplasm and protecting the plants from their deleterious effects [110]

In bryophyte, this mechanism was observed and described by Carginale et al. [36] and by Degola et al. [14] in *L. cruciata* exposed to cadmium. It was demonstrated that Cd accumulated in the vacuoles and that an increase in sulphur concentration was measured in this organelle. In addition, it was also found in *L. cruciata* cells that most of the intracellular Cd was bound to the thiol-rich compounds of similar weight, such as phytochelatins [14,36]. In a subsequent study by Maresca et al. [63], the authors demonstrated the application of PCs as specific biomarkers in the biomonitoring of a heavy-metal-polluted site. *L. cruciata* samples were collected in three sites with different degrees of pollution. The researchers found a strong correlation between the accumulated heavy metals in the *L. cruciata* thalli and the synthesized amount of PC2. Furthermore, the highest amount of PC2 was found in the most polluted site (Acerra), whilst no significant differences and correlations in PC3, PC4 and GSH were observed. In detail, PC2 was responsible for this trend (with an increase of 37% from Riccia to Naples and of 103% from Riccia to Acerra), while PC3 and PC4 were synthesized in incomparable amounts at the three sites. From the characterization and quantification of the thiol peptides, it was found that the PC3 and the PC4 were found to be low at all sites, while the PC2 showed increasing values from Riccia to Naples to Acerra.

Degola et al. [14] showed that in vitro exposure of *L. cruciata* to heavy metals for one week caused an increase in PC2, but also in PC3 and PC4. Interestingly, the only metals that were able to increase the activity of PC3 and PC4 in vitro were Cd, Fe and Zn, while As, Cu, Hg, Pb and Sb were not able to do so at any concentration. On the other hand, also in the moss *L. riparium*, following the treatment with Cd there was an increase of all three forms of phytochelatin, PC2, PC3 and PC4 [51]. Based on these outcomes, it is possible to hypothesize that the three phytochelatins take part in the heavy-metal-detoxification process, with different roles: PC3 and PC4 (alert) could be regarded as a “short term response”, as they are mainly produced in response to an unexpected stress situation to which the plant has not had time to adapt; in addition, such a response is triggered by a limited number of metals [63]. For a long time, it was thought that bryophytes cannot synthesize PCs under metal stress. However, Petraglia et al. [125] demonstrated that constitutively expressed and functional PCs are present in different bryophytes, in particular, in response to Cd. In the following years, other research supported this data. Although both GSH and PCs have a prominent role in metal detoxification, the main contributor of one or the other molecule to metal chelation is apparently different among species [20]. For example, with regard to Cd exposure, its detoxification is mainly driven by GSH for *L. riparium* [22], whereas PCs play central roles in *M. polymorpha* [126] and in *Nitella mucronate* [127]. In *Fontinalis antipyretica* no PCs, or only a negligible amount, were synthesized in response to heavy metal exposure [19]. In the same moss, there was an increase in GSH level and GSSG/GSH ratio in response to Cr exposure, but no dose-effect relationship could be observed. Moreover, two unknown thiol compounds were observed in mosses exposed to the highest Cr concentrations [90]. In a study by Sutter et al. [143], the authors compared the GSH and PCs content in three species of foliose liverworts (*Calypogeia arguta*, *Trichocolea tomentella*, *Scapania nemorea*) and the moss *Sphagnum fallax* after 10 days of exposure to Cd. The results indicated a depletion of GSH and a synthesis of PC2, PC3 and PC4 in the foliose liverworts after Cd exposure. On the other hand, they observed the accumulation of GSH but the absence of PCs in the moss species. These data support the hypothesis that mosses and liverworts might counteract heavy metal stresses in different manners. Comparing the response of *F antipyretica* and *Fontinalis dalecarlica* to the Cd exposed, it was shown that GSH increased in the first, in response to 10 days of exposure, but not in *F. dalecarlica*, indicating that the detoxification mechanism may be species-specific [128]. In *T. nepalense*, Choudhury et al. [38] showed that there was a higher accumulation of ascorbate and GSH under Pb, followed by Cr. The results showed that at a high concentration of metals, oxidative stress could be induced in moss cells, characterized by the generation of ROS and initiation of lipid peroxidation, which inhibited the major antioxidant metabolism [38]. In the same year, Panda and Choudhury [129] investigated the effect of Cr, Cu and Zn on nitrate reductase activity and responses to oxidative stress in the common moss *Polytrichum commune*, and observed the increase in the activity of some antioxidant enzymes, including glutathione reductase, after 24 and 48 h of metal treatment. The activation of the enzymes involved in the glutathione biosynthesis under heavy metal stress have been investigated in *P. patens* by Rother et al. [130]. The authors found an increase in the transcript for y-ECS and glutathione synthase (up to 7.9 and 3.2-fold 10 µM Cd, respectively). Furthermore, the activities of y-ECS and glutathione synthase were increased in a dose- and time-dependent manner. The authors also observed increases in the amount of cysteine, y-EC and GSH. However, no PCs were resolved through HPLC analysis in the Cd-exposed samples. The authors found a slight increase in O-acetylserine(thio)lyase (OAS-TL). Cysteine is a precursor for the synthesis of glutathione and phytochelatins. OAS-TL is responsible for the final step in cysteine biosynthesis, starting from O-acetilserine [144]. Similarly, Carfagna et al. [97] investigated the activity of the OAS-TL in *Scorpiurium circinatum* exposed to Cu, Zn, Pb, and Cd. After the treatments with 10^−4^ and 10^−5^ M of Cu, Zn, Pb and Cd for 24 h, the authors observed an induction of the activity of OAS-TL. Cd strongly induced the activity at the lowest concentration (10^−4^ M), while Pb and Cu did so at the highest concentration (10^−5^ M). These data indicate that cysteine biosynthesis is an early response to heavy metal stress that prepares the cells for the synthesis of glutathione and phytochelatins, to counteract further exposure.

### 2.9. Metallothioneins in Metal Detoxification

Metallothioneins (MTs) are low-molecular-weight, Cys-rich proteins found in all eukaryotic organisms, a few bacteria and recently in the moss *Physcomitrella patens* [145]. Although MTs have been discovered in plants over the last three decades, their precise physiological functions have not yet been fully clarified [146]. According to Hossain et al. [17], MTs are involved in (a) maintaining the homeostasis of essential transition metal ions, (b) the sequestration of toxic heavy metals, and (c) protection against intracellular oxidative perturbations. MTs are able to bind different metals by the formation of mercaptide bonds between the numerous Cys residues present in their structure [140]. Based on the Cys arrangement, it has been estimated that there are four groups of MTs in higher plants [147]; however, in bryophytes there is poor knowledge concerning this.

## 3. Conclusions

It is known that bryophytes have evolved different strategies to deal with environmental stresses, which are based on cellular rather than anatomical characteristics. A significant role in heavy metal tolerance and detoxification is played by vacuolar compartmentalization, which prevents the circulation of free metal ions into the cytoplasm, where they might interfere with key metabolic processes. The process of intracellular heavy-metal chelation and sequestration within the vacuole is accomplished by thiol compounds such as glutathione and phytochelatins. GSH, phytochelatins, antioxidant enzymatic system, and other organic small molecules seem to participate in counteracting the heavy metal stress in bryophytes. Moreover, only one study reported the expression of Cd-induced metallothionein-like genes in the model moss *P. patens*. To date, no metallothionein has been recovered and characterized in bryophytes which will expand our knowledge into the metal-tolerance mechanism in these organisms. The studies of this topic will be useful not only to understand how to enhance metal tolerance for environmental applications, but also to deepen understanding of the evolution of the early land plants.

## Data Availability

Not applicable.
